# Development and Validation of an Instrument for Assessing Patient Experience of Chronic Illness Care

**DOI:** 10.5334/ijic.2443

**Published:** 2016-08-31

**Authors:** José Joaquín Mira, Roberto Nuño-Solinís, Mercedes Guilabert-Mora, Olga Solas-Gaspar, Paloma Fernández-Cano, Maria Asunción González-Mestre, Joan Carlos Contel, Marío del Río-Cámara

**Affiliations:** 1 Clinical psychologist in Alicante-Sant Joan d’Alacant Health District, Consellería de Sanidad, Alicante, Professor, Miguel Hernández University, Elche, Alicante, REDISECC, Red de Servicios de Salud Orientados a Enfermedades Crónicas, Spain; 2 Head of Innovation at Deusto Business School Health, University of Deusto, Bilbao, Spain; 3 Department of Health Psychology Miguel Hernández University, Elche, Alicante, Spain; 4 International consultant on Health Policy and Management and associated researcher in NewHealth Foundation, Spain; 5 Public Policy at Merck Sharp & Dohme España (MSD), Madrid, Spain; 6 Head Expert Patient Programme Catalonia™, Chronic Care Programme, General Directorate of Health Planning, Barcelona, Spain; 7 Chronic Care Programme, Department of Health, Barcelona, Spain; 8 BIOEF, Basque Institute for Health Care Research and Innovation, Spain

**Keywords:** patient-centred care, integrated care, chronic illness, measurement, patient engagement

## Abstract

**Introduction:**

The experience of chronic patients with the care they receive, fuelled by the focus on patient-centeredness and the increasing evidence on its positive relation with other dimensions of quality, is being acknowledged as a key element in improving the quality of care. There are a dearth of accepted tools and metrics to assess patient experience from the patient’s perspective that have been adapted to the new chronic care context: continued, systemic, with multidisciplinary teams and new technologies.

**Methods:**

Development and validation of a scale conducting a literature review, expert panel, pilot and field studies with 356 chronic primary care patients, to assess content and face validities and reliability.

**Results:**

IEXPAC is an 11+1 item scale with adequate metric properties measured by Alpha Chronbach, Goodness of fit index, and satisfactory convergence validity around three factors named: productive interactions, new relational model and person’s self-management.

**Conclusions:**

IEXPAC allows measurement of the patient experience of chronic illness care. Together with other indicators, IEXPAC can determine the quality of care provided according to the Triple Aim framework, facilitating health systems reorientation towards integrated patient-centred care.

## Introduction

The prevalence of chronic conditions and multimorbidity is rising worldwide [1,2]. This growth has placed increasing demands on existing acute-oriented healthcare systems and has resulted in poor quality of care and deficient patient experiences as a consequence of the fragmentation and lack of coordination in the organisation and delivery of care for people living with chronic diseases [3].

Although many advances have been made in the treatment available to chronically ill patients, most patients are multimorbid with diverse clusters of chronic diseases, and current or potential complex health and social care needs. Management of these needs requires integral and proactive action and, in many cases, these complex patients do not receive the care recommended by evidence-based clinical practice guidelines [4].

Furthermore, there is also a lack of patients’ involvement and collaboration in the design and co-creation of health services, especially for those with chronic illnesses. As a consequence, the delivery of effective, high-quality chronic care requires a systemic transformation [5] that goes beyond merely adding new isolated interventions to the existing acute-focused healthcare system [6]. It requires patient engagement, widespread use of quality improvement methods and innovations in chronic care.

Two decades ago, Wagner et al. developed the Chronic Care Model [7], a framework for delivering care to patients with chronic conditions and for guiding quality improvement in chronic care. This model is focused on providing proactive, planned, integrated and patient-centred care. There is evidence that the Chronic Care Model improves clinical outcomes and experiences of chronically ill patients receiving care [8,9,10].

Cramm and collaborators have demonstrated that, over time, quality of care and changes therein translate into more positive experiences for patients with chronic conditions [11]. Therefore, patient experience can, if appropriately measured, indicate the quality of chronic illness care and can provide important information to improve quality of care, patient safety and clinical effectiveness [12].

Consistent with the Chronic Care Model, Glasgow et al. [13] developed the Patient Assessment of Chronic Illness Care scale (PACIC) to assess patient experience with chronic care delivery. This scale has been used internationally among patients with a variety of chronic health conditions, and has been adapted and validated in many countries. In a systematic review, Vrijhoef et al [14] identified it as the most applicable and relevant questionnaire for measuring the quality of integrated chronic care from the patient’s perspective. Recently, Singer et al [15,16] developed the Patient Perceptions of Integrated Care survey (PPIC), assessing a six-dimension model of integrated care. PACIC and PPIC focus on the experience between patients and the doctors and nurses who regularly provide their care. These instruments do not incorporate elements related to ICT developments in chronic care and do not directly assess the coordination between health care and social care providers.

Many new integrated care models are built to incorporate a patient’s narrative of needs, preferences and expectations [17,18], acknowledging the essential role they have in their own care and the need for truly patient-centred care [19]. However, there is a dearth of accepted metrics [20].

Our group previously developed IEMAC-ARCHO, a self-assessment tool of readiness for chronicity in healthcare organisations [21,22]. Subsequently, the need to develop an instrument to assess patient experience of chronic integrated care was identified for the following reasons:

– To incorporate new theories, frameworks and trends in health care, such as the Triple Aim [23], the narratives of ‘person-centred coordinated care’ [24] or coproduction approaches [25] that are emphasising the importance of patient experience;
– To incorporate a broad notion of integrated care, including social care and patient self-management;
– To include increasingly popular technological innovations that are transforming the interaction between patients and the system of care;
– To consider the epidemiological situation, characterised by high prevalence of chronic conditions and multimorbidity [26].
– To take into account the interaction with a team (or network) of providers instead of focusing on separate professionals (interactions with doctors, nurses, etc.) [27];
– To specifically address the concept of the “patient experience”, separating it from that of patient satisfaction considering the approaches and outcomes obtained by Michelle Beattie [28], Cramm [29,30] and Wensing [31]
– To complement the aforementioned tool with another that incorporates the patient perspective.

Therefore, the purpose of this paper is to describe the process of development and validation of a new tool to measure self-reported patient experience of integrated chronic care.

## Theory and methods

This is a design and validation study of a new instrument to assess the experience of patients with chronic conditions, who, because of their health status, have continuous interactions with social and health care professionals and services. The new tool is theoretically based on the Chronic Care Model and is inspired by patient-centred integrated care approaches.

The tool is intended to be used routinely to assess the patients experience of chronic illness care. For this reason, the following characteristics have been prioritised in its design [28]: small size (affordable), focused on the areas which patients consider important (appropriate), elements that support the processes of transformation and attention to chronicity (sensitive), orientation to what happens during the interaction with professionals (relevant), easy to understand (simple), a limited selection of elements (feasibility), suitable in any context (adaptable), and well-founded to ensure its psychometric properties (valid and reliable).

In this study, patient experience is defined as the information that the person facilitates on what has happened (to her) in her continued interaction with the health and social care professionals and services and on how she has lived that interaction and its outcomes. Meanwhile integrated care was conceptualised according to the Chronic Care Model and its subsequent adaptation by the WHO [3,7].

**Figure 1:**
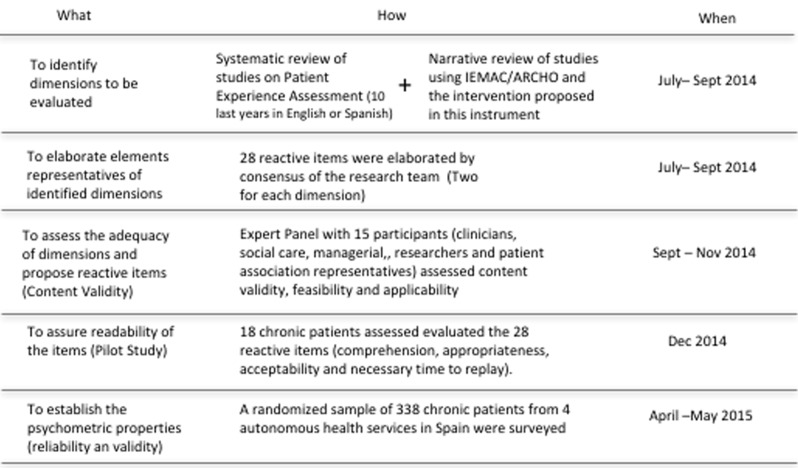
Summarises the steps followed in this study.

The steps followed in this study are shown in Figure 1.

### Literature search: Characteristics that should be analysed

The literature was reviewed to identify existing tools and their characteristics to evaluate patient experience with integrated chronic care. A scoping review was carried out using MEDLINE and Web-of-Knowledge. Only studies in English or Spanish published in the last ten years (until January 2015) were included considering the availability of a previous high quality systematic review conducted by Vrijhoef et al [14]. “Patient Experience” and “Patient Perceptions” with “Integrated Care” or “Chronic Care” were used as search terms. References from retrieved articles were examined to locate further studies. A total of 58 articles were found, their abstracts were revised and 18 of them were fully reviewed and incorporated as relevant sources of information. The results were initially analysed, structured and made available to all members of the research team.

### Selection and formulation of reactive items

Based on the previous results, the research team developed, by consensus, a pool of 28 reactive items. This set included a minimum of two items for each characteristic of care identified as relevant. Items were elaborated by the research team in successive work-team sessions considering: patient experience dimensions identified in the literature, as well as IEMAC/ARCHO [21,22] dimensions and interventions.

### Content validity: Expert panel

An expert panel was carried out from September to November 2014 using an online survey involving 15 professionals from primary care, public health, social services, management, quality and safety boards and research institutions. The selection of the participants was based on their knowledge and expertise, each having at least 15 years of experience in clinical or managerial positions. They were recruited by personal contact.

Experts evaluated: content validity (redundancies, absences, misleading questions), face validity (understanding, friendliness, adequacy, ordinal structure), relevance to justify items’ inclusion, adequacy of the type of response scale and the instrument as a whole. The results of their answers prompted some changes in the reactive items to be explored in this new instrument.

### Pre-test

Two pilot groups, each of 18 patients with chronic conditions, were conducted in December 2014. Patients were recruited through patient organisations for a variety of health conditions and had different characteristics (age, gender, socioeconomic). These patients evaluated the 28 reactive items of the questionnaire, regarding appropriateness, readability, acceptability and necessary time of response for each item, as well as two possible types of response scales. These patients considered the comprehension of this 28-item questionnaire as very good (4.8/5) as well as appropriate (4.6/5). The average time of response was 15 minutes. As a result, the wording of seven items was modified to improve understanding.

### Reliability and validity: Field study

To establish the psychometric properties of the instrument and select those items with the best behaviour, a field study was performed in April-May 2015 with the participation of 350 patients (sample size calculated for a p=q=0.50, 5% error and a level of significance for two queues of 0.05). These were patients older than 16 years of age, with at least one chronic disease, who visited general medicine or nursing consultants at 11 primary care centres of four regional health services in Spain (Catalonia, Madrid, Basque Country and Valencia). Among those who met the inclusion criteria, patients were recruited by the interviewer by random systematic cluster sampling with proportional allocation (K=3). The questionnaire was self-administered, and only at the request of the patient, applied by means of a personal interview. Patients interviewed were informed of the purpose of the study and informed consent was obtained. The demographic and clinical variables of the study were collected in a booklet of data collection (BDC) designed for this study. The interviewers received a briefing on the selection procedure of the patients to be interviewed and on the correct application of the questionnaire. Twelve patients declined to answer.

In the analysis, the ceiling-floor effects and the correlation of each item with the scale total score were considered, where values above 0.30 were acceptable [32]. To establish the construct validity, a preliminary study to determine the unidimensionality of the factors was conducted through the use of an exploratory factorial analysis (EFA) using principal components, with Varimax rotation of the resulting array. To remove factors, criteria were applied using Eigenvalues equal to 1 (calculating previously the statisticians Kaiser-Meyer-Olkin and the Bartlett’s test to determine the appropriateness of performed EFA). Factorial loads higher than 0.50 were considered as acceptable [33]. The internal consistency reliability of a first version of the instrument was calculated using the statistical Cronbach’s Alpha, assuming acceptable values equal to or greater than 0.70 [32,33].

Subsequently, a confirmatory factorial analysis (CFA) was carried out, using all data and a random selection (N=115) of data from patients receiving health care in different health services, to confirm the hypotheses concerning the underlying structure generated by the exploratory factorial analysis and to rate the ‘goodness of fit’. This analysis was performed using the three-factor model that was derived from EFA to verify that the isolated factors finally had not changed their structure and that statistics employed in the exploratory analysis remained satisfactory. To check the measurement model validity, the standardized root mean square residual (SRMR), the Jöreskog-Sörbom goodness of fit index (GFI), the normed fix index (NFI), and the comparative fix index (CFI) were used. Pearson correlations between factors were also calculated to check the factors’ independence.

The quality criteria for measurement properties of health status instruments proposed by Terwee et al. [34] were considered to assess the acceptability of the questionnaire’s elements.

The ability of the questionnaire to discriminate between isolated dimensions was tested by performing t-test, ANOVA or Chi-Square, because differences in scores were expected based on differences in care delivery.

### Ethical approval

The protocol of the study was approved by the Ethics Committee of the University Miguel Hernández, the institution who coordinated the study, and the Madrid Health Service Research Central Commission.

## Results

### Participants

Three hundred thirty-eight patients responded to the questionnaire (response rate 96.6%). Table 1 depicts their characteristics.

**Table 1:** Descriptive Characteristics Display.

Sample Description	N	%

Male	178	52.7
Female	160	47.3
Age (mean, SD)	66.5	14.1
16-30 years	7	2.1
31-45 years	24	7.1
46-65 years	102	30.2
≥66 years	205	60.7
Marital Status		
Single	46	13.6
Married	223	66.0
Widowed	69	20.4
Patients living alone	83	24.6
Educational level		
No studies	67	19.8
Basic studies	153	45.3
Grade education	50	14.8
University degree	68	20.1
Prescribed drugs (mean, SD)	4.1	3.8
Prescribed drugs grouped		
1-2 drugs	117	34.6
3-4 drugs	108	32.0
≥5 drugs	113	33.4
Who manages the medication		
The patient	306	90.5
The patient’s partner, another family member or caregiver	32	9.5
Disorders (a patient may suffer from more than one)		
Diabetes	72	21.3
Insulin-dependent diabetes	16	4.7
Arterial hypertension	190	56.2
COPD	68	20.1
Other cardiovascular diseases	97	28.7
Comorbidity		
1 disease	163	48.2
2 diseases	125	37.0
≥3 diseases	50	14.8
Months from the diagnosis		
Diabetes (mean, SD)	144.6	147.4
Arterial hypertension (mean, SD)	114.8	126.0
COPD (mean, SD)	116.9	137.0
Other cardiovascular diseases (mean, SD)	89.6	78.0
No hospitalisation in the last three years	197	58.3
No hospitalisation in the last year	253	74.9
No emergency visit in the last year	190	56.2
No general practitioner appointment in the last year	6	1.8
Hospitalisation in the last three years (mean, SD)	0.7	1.1
Hospitalisation in the last year (mean, SD)	0.3	0.6
Emergency visits in the last year (mean, SD)	0.9	1.8
General practitioner’ appointments last year (mean, SD)	7.4	11.5

### Items analysis

Seven items were ruled out for their ceiling-floor effects. The remaining 21 were included in the subsequent analysis, after verifying that there was acceptable variability in the answers of the patients.

The values of the Alpha of Cronbach were calculated by eliminating each item in an individualised way. No items were ruled out considering these data. The values of the correlations item-total ranged between 0.18 and 0.61. Three items with correlations inferior to 0.30 were ruled out before applying the technique of the exploratory factorial analysis.

### Explorative analysis of dimensionality and reliability: Exploratory factor analysis (EFA)

A first factorial solution, with 15 items, joined together in five first order factors, with a principal factor explaining the 51.6% of the variance, and four items with significant saturations in more than one factor. In the following exploratory factorial analysis, 11 items were included, each with factorial saturations higher than 0.5 and commonalities between 0.43 and 0.70. This factorial solution converged in three factors, explaining the 57.5% of the common variance (Table 2). Based on our observation, Factor 1, named “Productive Interactions”, refers to the characteristics and content of interactions between patients and professionals oriented to improve outcomes, for example the professionals who care for me listen to me and ask me about my needs/habits and preferences and they are concerned with my quality of life. Factor 2, named “New Relational Model”, refers to new forms of patient interaction with the health care system, through the internet or with peers. Factor 3, named “Patient Self-Management”, refers to the ability of individuals to manage their own care and improve their wellbeing based on professional-mediated interventions.

**Table 2:** Exploratory factorial analysis.

Item Abbreviation	Items	Communalities	Item-total Correlation	Alpha if item deleted	Mean*	SD	PI	NRM	PSM

P24	I review the adherence to my treatment and care plan with the professionals who care for me.	0.70	0.60	0.73	4.0	1.2	0,78		
P26	The professionals who care for me are concerned with my quality of life and I feel they are committed to my wellbeing.	0.64	0.53	0.73	4.1	1.2	0,77		
P22	Health and social care services are coordinated to improve my wellbeing and quality of life in my environment (family, neighbourhood, town).	0.62	0.9	0.74	4.0	1.2	0,77		
P9	The professionals who care for me listen to me and ask me about my needs, habits and preferences to adapt my treatment and care plan.	0.50	0.40	0.75	4.0	1.2	0,70		
P13	I can consult my clinical record, tests results, programmed visits and access to other services through the internet or the mobile app of my health service.	0.64	0.23	0.77	1.4	1.0		0.83	
P8	The professionals who care for me inform me about trustful webpages and internet forums that I can consult to better know my disease, its treatment and the consequences they may have on my life.	0.69	0.31	0.76	1.6	1.1		0.83	
P12	The professionals who care for me invite me to participate in patients groups to share information and experiences on how to care for ourselves and improve our health.	0.53	0.42	0.75	2.1	1.4		0.56	
P17	I’ve been able to agree with the professionals who care for me on specific objectives regarding diet, physical exercise and medication to get better control of my health problems.	0.61	0.47	0.74	3.6	1.4			0.74
P1	The professionals who care for me inform me about the health and social resources available in my neighbourhood or town that I can use to improve my health problems and take better care of myself.	0.46	0.31	0.77	3.1	1.6			0.68
P6	The professionals who care for me review with me all of the medication I take, how I take it and how it suits me.	0.49	0.41	0.75	4.0	1.3			0.51
P11	I feel that my confidence in my ability to take care of myself, manage my health problems and keep my autonomy has improved.	0.43	0.44	0.75	4.1	1.1			0.43
	Percentage of explained common variance (57%)						24%	17%	16%

N=338 *Scores 1 to 5 Extraction method: Principal Components. Rotation method: Varimax with Kaiser Normalization.

### Confirmatory analysis of dimensionality and analysis of reliability: Confirmatory factorial analysis (CFA)

**Figure 2:**
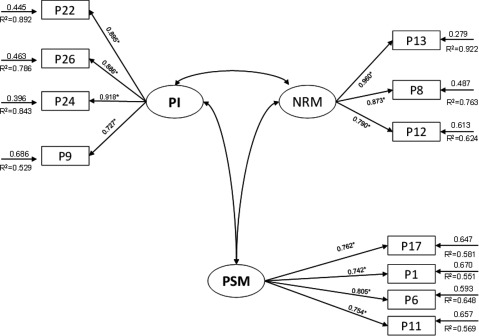
Optimised model of the questionnaire factorial structure based on confirmatory factor analysis from the validation study carried out.

The confirmatory factor analysis (CFA), in the second stage of the study, indicated an acceptable fit to the data. The estimates of the parameters and factor loadings of the model are shown in Table 3 and Figure 2. This figure also shows the optimised model of the questionnaire factorial structure based on confirmatory factor analysis.

**Table 3:** Confirmatory factorial index.

Evaluating model fit	Fit indices	A model is regarded as acceptable if:	CFA Index values	
			N= 338	N= 115

Absolute fit indices	SRMR	Value<0.08	0.05	0.06
	GFI	Value ≈1	0.96	0.89
Relative fit indices	NFI	Value ≈1	0.92	0.91
	CFI	0.9< Value <0.95	0.96	0.95

SRMR - Standardised Root Mean Square Residual; GFI- Jöreskog-Sörbom Fit Index-Goodness of Fix Index; NFI- Normed Fix Index; NNFI- Non Normed Fix Index; CFI- Comparative Fix Index; IFI- Incremental Fix Index.

### Composite reliability and convergent validity

The analysis of the convergent validity was satisfactory. All standardised loads were found to be significant for the respective factor and to be greater than 0.6. The average variance extracted was greater than 0.5 [35]. The composite reliability indexes were greater than 0.7 [36], indicating acceptable reliability for all factors (Table 4).

**Table 4:** Reliability, dimensionality, and convergent validity of each factor of the questionnaire.

Factor (abbreviation)	Items	Standardized loads^a^	

Productive Interactions (PI)	P22	0.895 (12.106)	
	P26	0.886 (11.907)	
	P24	0.918 (12.630)	
	P9	0.727 (8.838)	
			CR=0.918
			AVE=0.739
New Relational Model (NRM)	P13	0.960 (13.244)	
	P8	0.873 (11.406)	
	P12	0.790 (9.880)	
			CR=0.909
			AVE=0.769
Patient Self-management (PSM)	P17	0.762 (9.060)	
	P1	0.742 (8.735)	
	P6	0.805 (9.788)	
	P11	0.754 (8.928)	
			CR=0.950
			AVE=0.587

a Data represents Student *t*-test values and differences were significant at *P* = 0.05. b CR: composite reliability. c AVE: average variance extracted.

### Discriminant analysis

In the discriminant analysis, Table 5 shows the inter-correlations between the three factors identified in the analyses. The factors showed acceptable independence to each other. PI with PSM showed higher values.

**Table 5:** Inter-correlations between the latent factors.

	PI	NRM	PSM	Total Score

Productive Interactions (PI)	1.00	0.20	0.47	0.78
New relational model (NRM)		1.00	0.31	0.61
Person’s self-management (PSM)			1.00	0.82
Total Score				1.00

N=338 P<0.01

### Internal consistency

The value of the Alpha Chronbach was 0.76 for the whole scale (0.79 in factor 1, 0.56 in factor 2 and 0.63 in factor 3).

### Scale scores

The average punctuation in the IEXPAC-11 items was 3.1 points (SD 0.7, IC95% 3.0-3.2). The average punctuation in each factor was 4.0 points (SD 0.9) in factor one, 1.7 (SD 0.9) in factor two and 3.7 (SD 0.9) in factor three. Table 6 shows the results based on a set of variables.

**Table 6:** IEXPAC factors scores.

	N	%	PI	NRM	PSM

Male	178	52.7	3.96	1.58	3.63
Female	160	47.3	4.04	1.77	3.72
			*P* = 0.321	*P* = 0.033	*P* = 0.079
Age					
16-45	31	9.2	4.0	2.6	3.6
46-65	102	30.2	4.0	1.9	3.6
≥66	205	60.7	4.0	1.4	3.7
			*P* = 0.925	*P* = 0.001	*P* = 0.556
Educational level					
No studies	67	19.8	4,00		
	1.28	3.52			
Basic studies	153	45.3	4,00	1.48	3.72
Grade education	50	14.8	4,12	1.96	3.65
University degree	68	20.1	3,91	2.30	3.75
			*P* = 0.696	*P* = 0.000	*P* = 0.394
Who they live with					
Alone	83	24.6	4,04	1.51	3.63
In family	252	74.6	4,00	1.74	3.69
			*P* = 0.467	*P* = 0.021	*P* = 0.463
Chronic diseases					
1	163	48.2	4.2	1.8	3.7
2	125	37.0	3.8	1.6	3.6
≥3	50	14.8	3.8	1.5	3.6
			*P* = 0.001	*P* = 0.068	*P* = 0.707
Prescribed drugs					
1-2	117	34.6	4.1	1.9	3.6
3-4	108	32.0	3.9	1.6	3.8
≥5	113	33.4	4.0	1.5	3.7
			*P* = 0.306	*P* = 0.001	*P* = 0.351
					
Number of hospitalisations in last 3 years					
0	197	58.3	4.0	1.7	3.6
1-2	119	35.2	4.0	1.6	3.7
3 or more	22	6.5	4.1	1.6	3.9
			*P* = 0.947	*P* = 0.657	*P* = 0.401
					
Emergency visits in the last year					
0	191	56.5	4.1	1.7	3.7
1-2	116	34.3	3.9	1.6	3.6
3 or more	31	9.2	3.8	1.5	3.6
			*P* = 0.179	*P* = 0.281	*P* = 0.468
General practitioner appointments in the last year					
0	9	2.1			
1-3	133	39.3	4.0	1.9	3.7
4-5	51	15.1	3.8	1.6	3.4
6 or more	145	42.9	4.1	1.4	3.8
			*P* = 0.255	*P* = 0.001	*P* = 0.085
					
Autonomous Health Service					
1	84	24.9	4.1	2.6	3.9
2	84	24.9	4.3	1.4	3.3
3	85	25.1	3.5	1.3	3.6
4	85	25.1	4.1	1.4	3.9
			*P* = 0.001	*P* = 0.001	*P* = 0.001

The new tool was named IEXPAC, Instrument for Evaluation of the Experience of Chronic Patients (available online at http://www.iemac.es/iexpac/). Additionally, an item was included for specific cases when a patient is hospitalised, to check the continuity of care during discharge and once the patient returns home. This item (number 12) is not included in the scale aggregate rating. The average score for this item was 2.5 (SD 1.7).

## Discussion

The developed scale is an instrument to obtain a reliable and valid measure of the experience of patients with chronic conditions during their interaction with the system of care. IEXPAC has a condensed set of measured items so that it can be used routinely and systematically in care services for assessing whether patients perceive is receiving integrated care, have a positive relationship with the set of professionals which usually interacts with, feels abler to look after their health and, are involved in new ways of non-face-to face interactions.

Specifically, IEXPAC assesses patient experience in accordance with the Triple Aim framework. Triple Aim shows the importance of the “experience of care”, an inconsistently measured dimension. Most health systems do not regularly assess the experience of care. This instrument could be used online (http://iexpac.es) paper-and-pencil or phone to assess patient experience of care in several contexts such as a health centre, health district or health service.

Patient experience represents a unique encompassing dimension that is challenging to measure. Lacking a widely accepted definition [37], we have rooted the development of this scale in the Chronic Care Model theoretical framework, enriched with approaches coming from patient-centred care [17,18] and service coproduction theories [25]. The relationship between patient experience and quality of care is not consistent in the literature. Major studies have rendered different, even opposite, results [12,38,39], whose likely explanation has been analysed by Manary [40]. In many contexts, the established patient satisfaction terminology is being substituted for the rather new ‘patient experience’, as if they can be used interchangeably. Patient experience with chronic care, as captured with the IEXPAC scale, however, differs significantly from the traditional patient satisfaction with episodes of care. This is why, in developing IEXPAC, we put emphasis on clarifying and delimiting the concept we want to measure, as formulated in our definition of patient experience. In the past, the concept of satisfaction has been used in a bilateral way, capturing the interaction of a patient with a single professional or organization. New measures should be focused on gauging experience with more comprehensive and complex provision models where different organizations are working collaboratively to provide patient-centred care.

The IEXPAC scale has several strengths from an integrated care point of view. First, it assesses experience of care beyond punctual contact, episode or specific setting. It is expected to capture a continuous experience over six months. Second, it considers the health professionals as a team, not solely focused on individual or isolated interaction with physicians, nurses or other staff. In countries like Spain, care delivery is not only focused on “doctors”, but on a comprehensive team, where different primary care professionals interact with patients and different providers from hospitals, mental health networks, long term care facilities or social care organisations. Other authors, such as Walker et al have also acknowledged that patients “highly value a sense of all members of the care team being on the same page”. Third, the scale captures the relationship between the patient and a system of care, where self-management and social care are also relevant. Fourth, the scale incorporates an active patient role, having a clear orientation towards improving outcomes by means of patients and professionals working together (coproduction). Fifth, IEXPAC is aligned with new evaluative frameworks of population health management based on the Triple Aim vision.

The psychometric analysis of IEXPAC renders three independent factors with items that converge around concepts: productive interactions, new relational model and patient self-management, all with literature supporting their adequacy and soundness [41].

This instrument also has some limitations that should be considered. There is still no data to support whether improvements in the scale ratings are related to better clinical or health-related quality of life outcomes. Furthermore, as patients with different chronic diseases or patients in different settings have distinct experiences with chronic illness care, there may be a need for specific scales for certain chronic diseases or types of complex chronic patients, such as those in home care programmes and those who are assisted by caregivers. Finally, the way the scale is formulated does not allow to attribute responsibilities to a specific care provider at individual level, only to a team of providers or “system of care”.

To the best of our knowledge, most national health systems are not capturing the integrated care experience of patients with chronic conditions in a regular, holistic and systematic way. Most countries have a range of measures related to health outcomes and cost or utilization of services, typically included in most national or regional outcome frameworks. There is appropriate to also develop experience of care measures and incorporate them into national and regional-level integrated care models. There is a promising future for these metrics through the commissioning by health and social care authorities and through performance assessments. Relevant initiatives are expected to appear in the coming years in this field of knowledge [42,43]. For example, the area 4 (Ensuring that people have a positive experience of care) in the English NHS Outcome Framework 2015/2016 [44], may include the assessment of experience of care from an integrated care perspective. Tools like IEXPAC should contribute to this aim.

## Conclusions

IEXPAC scale measures the experience of patients with chronic conditions in their continued interaction with health professionals and services in regular practice. It can ascertain the quality of care experienced by patients, contributing to the ‘experience of care’ axis of the Triple Aim, and facilitate the adoption of patient-centred care approaches by health and social care organisations.

Measurement of patient experience may also facilitate the reorientation towards patient-centred care. Presently, with numerous processes of service integration being deployed, this might be of particular importance. It is necessary to generate results that consolidate this type of measurement, showing its correlation with other outcome indicators whose relevance and usefulness are widely accepted in the literature.

Although IEXPAC has yet to prove it can be used in regular practice, it seems that new metrics like this will be welcomed and possibly incorporated into regular performance assessments or commissioning processes of health and social care.
